# Joint Lp-Norm and L_2,1_-Norm Constrained Graph Laplacian PCA for Robust Tumor Sample Clustering and Gene Network Module Discovery

**DOI:** 10.3389/fgene.2021.621317

**Published:** 2021-02-23

**Authors:** Xiang-Zhen Kong, Yu Song, Jin-Xing Liu, Chun-Hou Zheng, Sha-Sha Yuan, Juan Wang, Ling-Yun Dai

**Affiliations:** School of Computer Science, Qufu Normal University, Rizhao, China

**Keywords:** Lp-norm, graph regularization, sparse constraint, principal component analysis, tumor clustering, gene network modules, L_2, 1_-norm

## Abstract

The dimensionality reduction method accompanied by different norm constraints plays an important role in mining useful information from large-scale gene expression data. In this article, a novel method named Lp-norm and L_2,1_-norm constrained graph Laplacian principal component analysis (PL21GPCA) based on traditional principal component analysis (PCA) is proposed for robust tumor sample clustering and gene network module discovery. Three aspects are highlighted in the PL21GPCA method. First, to degrade the high sensitivity to outliers and noise, the non-convex proximal Lp-norm (0 < *p* < 1)constraint is applied on the loss function. Second, to enhance the sparsity of gene expression in cancer samples, the L_2_,_1_-norm constraint is used on one of the regularization terms. Third, to retain the geometric structure of the data, we introduce the graph Laplacian regularization item to the PL21GPCA optimization model. Extensive experiments on five gene expression datasets, including one benchmark dataset, two single-cancer datasets from The Cancer Genome Atlas (TCGA), and two integrated datasets of multiple cancers from TCGA, are performed to validate the effectiveness of our method. The experimental results demonstrate that the PL21GPCA method performs better than many other methods in terms of tumor sample clustering. Additionally, this method is used to discover the gene network modules for the purpose of finding key genes that may be associated with some cancers.

## Introduction

High-throughput sequencing technologies, including genome-wide measurements, have enabled large-scale gene expression profiles to accumulate faster (Goodwin et al., 2016). It is of great significance to obtain useful information from these data. Reliable and precise identification of cancer types and obtaining key pathogenic genes are very important for cancer diagnosis and treatment (Koboldt et al., 2012). Generally, gene expression data have a typical characteristic of “high dimension, low sample” size (West, 2003), which is a challenge for most traditional statistical methods. Too many variables and some uncorrelated noise variables in the gene expression data may all have a negative effect on the tumor clustering performance regardless of whether supervised or unsupervised clustering methods are used. Despite these problems, many researchers have demonstrated the effectiveness of tumor-type identification and feature selection by leveraging many machine learning algorithms (Hochreiter et al., 2010; Lee et al., 2010; Liu J. X. et al., 2015; Bunte et al., 2016; Kong et al., 2017; Wang et al., 2017; Chen et al., 2019). Among them, algorithms based on principal component analysis (PCA) (Collins, 2002; Jolliffe, 2002) have been widely used to process gene expression data successfully (Liu et al., 2013; Liu J. X. et al., 2015; Wang et al., 2017; Feng et al., 2019) for dimension reduction and denoising. However, PCA-based algorithms, including sparse principal component analysis (SPCA) (Zou et al., 2006; Shen and Huang, 2008; Journee et al., 2010; Liu et al., 2016; Feng et al., 2019) and robust principal component analysis (RPCA) (Candès et al., 2009; Liu et al., 2013; Liu J. X. et al., 2015; Wang et al., 2017), mainly deal with data that lie in a linear data manifold (Jiang et al., 2013). Many methods that can handle data lying in a non-linear manifold have been proposed, such as locality preserving projections (LPP) (He et al., 2005), locally linear embedding (LLE) (Roweis and Saul, 2000), local tangent space alignment (Zhang and Zha, 2002), Laplacian eigenmap (LE) (Belkin and Niyogi, 2002, 2003; Spielman, 2007) and latent variable model (LELVM) (Keyhanian and Nasersharif, 2015). The purpose of these non-linear dimensionality reduction techniques is to find a representation of points (samples) in a low-dimensional space, in which all points (samples) still maintain the similarity in the original high-dimensional space.

In recent years, optimization models that combine linear and non-linear dimensionality reduction methods, especially graph Laplacian embedding, have demonstrated their effectiveness. Liu et al. (2017) constructed a graph Laplacian matrix for semisupervised feature extraction. Cai et al. (2011) proposed a method named graph regularized non-negative matrix factorization (GNMF), which combined graph structure and non-negative matrix factorization for an improved compact representation of the original data. Jiang et al. (2013) developed graph-Laplacian PCA (gLPCA), which sought a low-dimensional representation of image data with significant improvement in clustering and image reconstruction by incorporating graph structures and PCA. Feng et al. (2017) employed pgLPCA based on graph Laplacian regularization and Lp-norm for feature selection and tumor clustering. Wang et al. (2019a) used Laplacian regularized low-rank representation (LLRR), which considers the intrinsic geometric structure of gene expression data to cluster the tumor samples. In addition, many methods benefit from norm constraints. For example, Journee et al. (2010) employed the L_0_-norm constraint based on PCA to stress the sparse expression of genes in samples. The L_1_-norm (Tibshirani, 1996) was introduced as the regularization function in sparse singular value decomposition (SSVD) (Lee et al., 2010; Kong et al., 2017) and the mix-norm optimization model proposed by Wang et al. (2019b). Feng et al. (2016) employed the L_1/2_-norm constraint in their model to select characteristic genes. However, there remain some facets to be improved: for example, the robustness of the algorithm should be enhanced further, and the sparse representation of the data should be highlighted. For these purposes, the Lp-norm (Chartrand, 2012; Nie et al., 2013; Feng et al., 2017; Kong et al., 2017) constraint was used in the optimization model to degrade the sensitivity of outliers of the data. The L_2,1_-norm (Xiang et al., 2012; Yang et al., 2012) constraint was used by Liu et al. (2017) and Wang et al. (2019b) to generate the row sparsity.

Motivated by the literature mentioned above, especially (Tibshirani, 1996; Chartrand, 2012; Xiang et al., 2012; Nie et al., 2013; Feng et al., 2017; Kong et al., 2017), we propose a new method named PL21GPCA incorporating traditional PCA, graph Laplacian embedding and different norm constraints on the loss function and the regularization function for robust tumor sample clustering and gene network module discovery. Five gene expression datasets, including one benchmark dataset, two single-cancer datasets from The Cancer Genome Atlas (TCGA), and two integrated datasets of multiple cancers from TCGA, are used to evaluate the effectiveness of our method. The experimental results demonstrate that the PL21GPCA method outperforms many existing methods in terms of tumor sample clustering. Additionally, this method is employed to discover gene network modules to identify the key genes with close relationships to some cancers.

We organize the rest of this paper as follows. Section “Related Works” provides the related works containing the non-convex proximal Lp-norm, L_2,1_-norm and graph regularized PCA. The optimization model of PL21GPCA is presented, and the solution procedure is detailed in section “Methodology.” Section “Experiments and Discussion” presents the parameter selections, experimental results and some discussions. The tumor sample clustering and gene network analysis are also described in this section. In Section “Conclusion and Suggestions,” we present the conclusion for this article and propose some suggestions for future research.

## Related Works

### Definitions of the Proximal Lp-Norm and L_2,1_-Norm

Let _*X∈R^p×n^*_ be a data matrix, the proximal Lp-norm of _*X*_ is defined as follows:

(1) $$∥X{∥}_{p}={\left(\sum_{i}^{p}\sum_{j}^{n}|x_{i\,j}{|}^{p}\right)}^{\frac{1}{p}}\left(0>p>1\right)$$

The Lp-norm with 0 < *p* < 1 is a function with three typical characteristics: globally non-differentiable, non-convex, and non-smooth (Chartrand, 2012; Zhang et al., 2015). Many researchers have made suggestions to deal with Lp-norm (0 < *p* < 1) minimization (Chartrand, 2012; Guo et al., 2013; Qin et al., 2013). Since Lp-norm minimization can result in a sparser solution than the L_1_-norm and perform better in terms of robustness to outliers than the L_2_-norm in a sense, we use it to constrain the loss function of the PL21GPCA optimization model. The generalized shrinkage operation proposed by Chartrand (2012) is adopted to solve the function effectively in our method.

The L_2,1_-norm of matrix _*X*_ is as follows:

(2) $${∥X∥}_{2,1}=\sum_{i=1}^{p}\sqrt{\sum_{j=1}^{n}x_{i\,j}^{2}}=\sum_{i=1}^{p}{∥x_{i}∥}_{2}$$

where *x*_*i*_ (corresponding to feature *i*) is the _*i*_th row of matrix _*X*_. Yang et al. (2012) provided an intuitive explanation of the L_2,1_-norm in the literature. To solve the *L*_*2,1*_-norm, we can compute the *L*_*2*_-norm of each row of _*X*_ first, record it as a vector *b*(*X*) = (∥*x*_1_∥_2_,∥*x*_2_∥_2_,…,∥*x*_*p*_∥_2_), and then compute the *L*_*1*_-norm of vector *b*(*X*). The components of vector *b* indicate the importance of each feature. The L_2,1_-norm favors obtaining a small number of non-zero rows in matrix _*X*_, and then feature selection will be achieved.

### PCA and Graph Laplacian Embedding

#### Principal Component Analysis (PCA)

Let *X* = (*x*_1_,⋯,*x*_*n*_) ∈ *R*^*p*×*n*^ (*p*≫*n*) be a matrix whose rows represent genes and columns represent samples. PCA is usually used to find the optimal principal directions *V*^*T*^ = (*v*_1_,⋯,*v*_*n*_) ∈ *R*^*k*×*n*^ (*V*^*T*^*V* = *I*) that define the low-dimensional (*k*-dim) subspace. And the projected data points in the low subspace *V*can be denoted as the elements of the matrix *U*_*p*×*k*_ = (*u*_1_,⋯,*u*_*k*_) ∈ *R*^*p*×*k*^. The traditional PCA finds *U*and *V* with the squared Frobenius norm:

(3) $$\underset{U,V}{\arg}\min{∥{\text{X-UV}}^{T}∥}_{F}^{2}\,\,s.t.\,V^{T}\,V=I$$

In our optimization model, the proximal Lp-norm ∥g∥_*p*_ (0 < *p* < 1) (Chartrand, 2012; Nie et al., 2013; Feng et al., 2017) is used instead of the traditional quadratic loss function ∥g∥_*F*_ to reduce the influence of outliers and noise. PCA naturally relates closely to the classic clustering means known as K-means (Ding and He, 2004). The optimal principal components contained in matrix *V* provide the solution of the K-means clustering method. It inspired us to combine PCA with Laplacian embedding, whose principal purpose is also clustering.

#### Graph Laplacian Embedding

Principal component analysis can find an approximate set of basis vectors in the case where data usually lie in a linear manifold (Jiang et al., 2013). In consideration of the local invariance of the intrinsic geometric structure of the data distribution, graph Laplacian embedding is a popular method among recent studies in non-linear manifold learning theory (Belkin and Niyogi, 2002, 2003; Spielman, 2007). The assumption of local invariance is that if two points (samples) are close in the intrinsic geometry of the original data distribution, the representations of these two points (samples) in the new coordinate are also close to each other. The local geometric structure can be modeled through a nearest neighbor graph on a scatter of data points. Given the data matrix *X* = (*x*_1_,⋯,*x*_*n*_) ∈ *R*^*p*×*n*^, *x*_*i*_(*i* = 1,⋯,*n*)can be regarded as one data point (one vertex in the graph). For each data point *x*_*i*_, we find its *k*′ nearest neighbors and put edges between *x*_*i*_ and its neighbors. Then, a graph with _*n*_ vertices can be constructed, on which the weight matrix _*W∈R^n×n^*_ is defined. *w*_*ij*_ is the weight between vertices *x*_*i*_ and *x*_*j*_, it is used to measure the closeness of two points *x*_*i*_ and *x*_*j*_, and it is a symmetric similarity matrix. There are three popular choices defining the weight matrix on the graph: heat kernel weighting, 0–1 weighting, and dot-product weighting. If nodes _*i*_ and _*j*_ are connected, using heat kernel weighting, $w_{i\,j}=e^{\frac{{∥x_{i}-x_{j}∥}^{2}}{\sigma }}$, *w*_*ij*_ = 1 using 0–1 weighting and $w_{i\,j}=w_{i}^{T}\,w_{j}$ using dot-product weighting. The different similarity measures are suitable for different situations. Detailed information about the different weighting schemes can be found in the literature (Cai et al., 2011).

Let *Z*^*T*^ = (*z*_1_,*z*_2_,⋯,*z*_*n*_) ∈ *R*^*k*×*n*^ represent the _*n*_data points in the *k*-dim embedding coordinates *Z*^*T*^ = (*v*_1_,⋯,*v*_*n*_) ∈ *R*^*k*×*n*^ (*Z*^*T*^*V* = *I*), i.e., the representation of _*x_i*_ in the new low-dimensional basis is *z*_*i*_ = [*v*_*i*1_,⋯,*v*_*ik*_]. The “dissimilarity” of the two data points in the low basis can be measured by the Euclidean distance or the divergence distance. The Euclidean distance is adopted in our method. Define the “dissimilarity” of the two points in the low basis as *d*(*z*_*i*_,*z*_*j*_) = ∥*z*_*i*_−*z*_*j*_∥^2^, combined with the weight matrix _*W*_, and the smoothness of the low-dimensional representation can be measured by minimizing:

$$S=\frac{1}{2}\,\sum_{i,j=1}^{n}{∥z_{i}-z_{j}∥}^{2}\,w_{i\,j}$$

$$=\sum_{i=1}^{n}z_{i}^{T}\,z_{i}\,D_{i\,i}-\sum_{i,j=1}^{n}z_{i}^{T}\,z_{j}\,w_{i\,j}$$

(4) $$=T\,r\,\left(V^{T}\,D\,V\right)-T\,r\,\left(V^{T}\,W\,V\right)=T\,r\,\left(V^{T}\,L\,V\right)$$

where _*Tr(•)*_is the trace of a matrix, _*D=diag(d_1,⋯,d_n)*_is a diagonal matrix, and ${}_{d_{i}=\sum_{j=1}^{n}w_{i\,j}}$. We call the _*L=D–W*_ the Laplacian matrix (Spielman, 2007).

## Methodology

**FIGURE 1:**
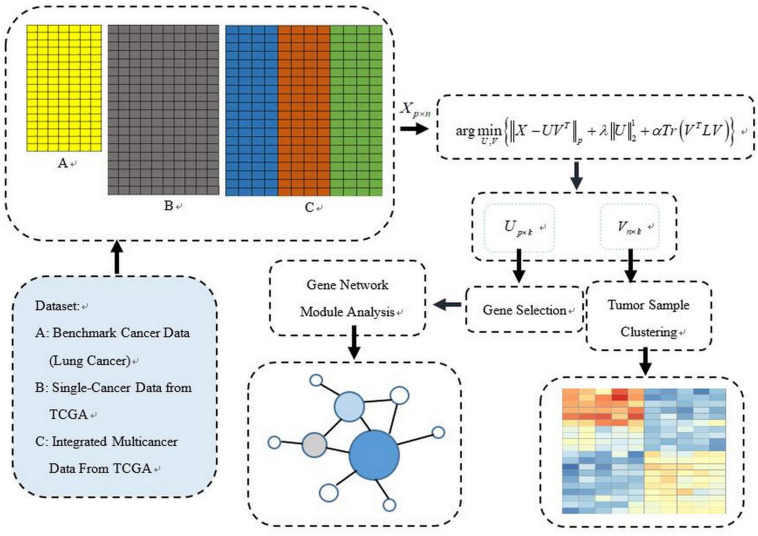
The general schematic framework of the PL21GPCA methodology.

The PL21GPCA procedure is presented in this section. Figure 1 illustrates our general research framework. In brief, our work includes two steps. The first is obtaining the optimal projected matrix *U*_*p×k*_ and the principal directions matrix *V*_*k×n*_ via PL21GPCA. The second is to evaluate the validity of PL21GPCA. In this step, based on the principal directions matrix *V*_*k×n*_ obtained by PL21GPCA, the classic clustering method K-means is employed for tumor sample clustering. According to the projected matrix *U*_*p×k*_, the differentially expressed genes are selected to carry out gene network analysis to find the key genes with close relationships to some cancers.

To summarize, three aspects are highlighted in our method:

(1) To reduce the influence of outliers and noise, the non-convex proximal Lp-norm ∥g∥_*p*_(0 < *p* < 1) is used on the loss function, which could improve the robustness of the optimization model effectively compared with the other constraints.
(2) To enhance the sparsity of gene expression in cancer samples, the L_2,1_-norm is used on the projected matrix *U*_*p× k*_.
(3) To retain the intrinsic geometric structure of the data points (samples), the graph regularization item is recommended in the optimization model.

Assume the input matrix *X* = (*x*_1_,⋯,*x*_*n*_) ∈ *R*^*p*×*n*^(*p*≫*n*), which denotes _*p*_ genes’ expression in _*n*_ samples. Our goal is to find the optimal low-dimensional (_*k*_-dim) subspace denoted as *Z*^*T*^ = (*v*_1_,⋯,*v*_*n*_) ∈ *R*^*k*×*n*^ (*Z*^*T*^*V* = *I*) and the projected matrix *U*_*p*×*k*_ = (*u*_1_,⋯,*u*_*k*_) ∈ *R*^*p*×*k*^ in the low subspace. The traditional PCA finds *U*and *V* with the squared Frobenius norm in the solution. In our optimization model, the proximal Lp-norm ∥g∥_*p*_(0 < *p* < 1) (Chartrand, 2012; Nie et al., 2013; Feng et al., 2017) replaces the traditional quadratic loss function ∥g∥_*F*_ to reduce the influence of outliers and noise. The L2,1-norm is used on one of the regularization terms to enhance the sparse gene expression in cancer samples. The graph Laplacian regularization item emphasizing the local invariance of the intrinsic geometric structure is recommended in the optimization model.

The objective function of this method is designed as follows:

(5) $$\begin{array}{c} \underset{U,V}{\arg\min}\left\{{∥{\text{X-UV}}^{T}∥}_{p}+\lambda \,{∥U∥}_{2}^{1}+\alpha \,T\,r\,\left(V^{T}\,L\,V\right)\right\}\\ s.t.\,V^{T}\,V=I,\,0>p>1,\,\lambda <0,\,\alpha <0 \end{array}$$

Clearly, the objective function is somewhat intractable because it is non-convex and non-smooth. We adopt the augmented Lagrangian multiplier (ALM) (Hestenes, 1969; Bertsekas, 1982; Spielman, 2007; Lin et al., 2010) to address this optimization problem. Researchers have proven that the ALM algorithm possesses Q-linear convergence properties under some conditions (Bertsekas, 1982).

When using the ALM method to obtain the optimal solution of (5), we replace *X*−*UZ*^*T*^ with _*E*_. Eq. (5) can be equivalently written as:

(6) $$\begin{array}{c} \underset{E,U,V}{\arg\min}\left\{{∥E∥}_{p}+\lambda \,{∥U∥}_{2}^{1}+\alpha \,T\,r\,\left(V^{T}\,L\,V\right)\right\}\\ s.t.\text{E-X}+U\,V^{T}=0,\,V^{T}\,V=I \end{array}$$

According to the ALM method, eq. (6) is equivalent to minimizing:

(7) $$\begin{array}{c} L_{\mu ,Y}\,\left(E,U,V\right)={∥E∥}_{p}+\frac{\mu }{2}\,{∥\text{E-X}+U\,V^{T}+\frac{Y}{\mu }∥}_{F}^{2}\\ +\lambda \,{∥U∥}_{2}^{1}+\alpha \,T\,r\,\left(V^{T}\,L\,V\right), \end{array}$$

where _*Y*_ is the Lagrangian multiplier, and _μ_ is the step size of the update rule. In (7), there are three variables to be solved. The alternating direction method (ADM) (Gabay and Mercier, 1976) is adopted to tackle this thorny problem because the equation with only one variable is easily solved when the others are fixed. By this means, (7) naturally results in three subproblems.

Problem 1: When _*U*_ and _*V*_ are fixed, (7) is written as follows:

(8) $$L_{\mu ,Y}\,\left(E,U,V\right)={∥E∥}_{p}+\frac{\mu }{2}\,{∥\text{E-X}+U\,V^{T}+\frac{Y}{\mu }∥}_{F}^{2}$$

where 0>p>1. Eq. (8) can be solved by the proximal shrink operator denoted as follows:

(9) $$s\,h\,r\,i\,n\,k_{p}\,\left(t,\delta \right):=\max\left\{0,\left|t\right|-\delta \,{\left|t\right|}^{p-1}\right\}\,\frac{t}{\left|t\right|}$$

Let $t=X-U\,V^{T}-\frac{Y}{\mu }$, $\delta =\frac{1}{\mu }$. Then, according to the shrinkage operation (soft thresholding) proposed by Chartrand (2012), _*E*_ is updated as:

(10) $$E^{r+1}=s\,h\,r\,i\,n\,k_{p}\,\left\{X-U^{r}\,{\left(V^{t}\right)}^{T}-\frac{Y^{r}}{\mu^{r}},\frac{1}{\mu^{r}}\right\}$$

Problem 2: When _*E*_ and _*V*_ are fixed, (7) is simplified as follows:

(11) $$L_{\mu ,Y}\,\left(E,U,V\right)=\frac{\mu }{2}\,{∥\text{E-X}+U\,V^{T}+\frac{Y}{\mu }∥}_{F}^{2}+\lambda \,{∥U∥}_{2}^{1}$$

To simplify (11), let $H=X-E-\frac{Y}{\mu }$. Then, (11) is written as:

(12) $$L_{\mu ,Y}\,\left(E,U,V\right)=\frac{\mu }{2}\,{∥U\,V^{T}-H∥}_{F}^{2}+\lambda \,{∥U∥}_{2}^{1}$$

The partial derivatives of _*L*_ with respect to _*U*_are:

(13) $$\frac{\partial L}{\partial U}=\mu \,\left(U\,V^{T}-H\right)\,V+2\,\lambda \,Q\,U$$

where _*Q∈R^p×p^*_ is a diagonal matrix with $q_{i,i}=\frac{1}{{∥U_{\left(i,:\right)}∥}_{2}}\left(i=1,\cdots ,p\right)$ (Xiang et al., 2012). Letting (13) be equal to 0, the following update rule for _*U*_ is then obtained:

(14) $$U^{r+1}={\left(I+\frac{2\,\lambda }{\mu^{r}}\,Q^{r}\right)}^{-1}\,H^{r}\,V^{r}$$

To simplify (14), let $A^{r}={\left(I+\frac{2\,\lambda }{\mu^{r}}\,Q^{r}\right)}^{-1}$, and then (14) is written as:

(15) $$U^{r+1}=A^{r}\,H^{r}\,V^{r}$$

Problem 3: When _*E*_ and *W*are fixed, (7) is simplified as follows:

(16) $$L_{\mu ,Y}\,\left(E,U,V\right)=\frac{\mu }{2}\,{∥\text{E-X}+U\,V^{T}+\frac{Y}{\mu }∥}_{F}^{2}+\alpha \,T\,r\,\left(V^{T}\,L\,V\right)$$

With respect to the settings $H=X-E-\frac{Y}{\mu }$, (16) can be written equivalently as:

(17) $$\begin{array}{ccc} L_{\mu }\,\left(E,U,V\right) & = & \frac{\mu }{2}\,{∥U\,V^{T}-H∥}_{F}^{2}+\alpha \,T\,r\,\left(V^{T}\,L\,V\right)\\ & = & \frac{\mu }{2}\,T\,r\,\left(\left(U\,V^{T}-H\right)\,{\left(U\,V^{T}-H\right)}^{T}\right)+\alpha \,T\,r\,\left(V^{T}\,L\,V\right) \end{array}$$

Based on (17), _*V*_is found by minimizing:

(18) $$V=\underset{V}{\arg}\min T\,r\,\left(V^{T}\,\left(\frac{\alpha }{\mu }\,{\text{L-H}}^{T}\,A\,H\right)\,V\right)$$

Therefore, _*V^r+1^*_ can be obtained as follows:

(19) $$V^{r+1}=\left(v_{1},\ldots ,v_{k}\right)$$

where (*v*_1_,…,*v*_*k*_) are the _*k*_eigenvectors corresponding to the smallest _*k*_ eigenvalues of the matrix $\frac{\alpha }{\mu }\,{\text{L-H}}^{T}\,A\,H$. Thus, based on the ALM, ADM and the shrinkage operation, the solution to solve the optimization model described in (5) is shown in Algorithm 1. In the optimization model, there are six parameters _*k*_, _*p*_, _λ_, _α_, _ρ_, _μ_ to be pre-determined, among them. As the parameters used to control the step size in the update rule of AML, we set μ = 10^−2^ and ρ = 1.2 for all gene expression datasets experiments (Feng et al., 2016). The parameter _*k*_ is determined refering to the number of prior categories of each dataset. For the three essential parameters _*p*_, _λ_, _α_, to be determined in (5), we choose them corresponding to different situations for the best clustering performance through extensive experiments. Different parameters are chosen for different datasets. Detailed parameter selections and discussions are described in section “Experiments and Discussion.”

**ALGORITHM 1:** The solution to optimized (5).

**Input:**
Gene expression data matrix: _*X_p×n*_,
Parameters: _*k*_, _*p*_, _λ_, _α_, _ρ_, _μ_
**Output:**
_*U_p×k*_, _*V_n×k*_
**Initialize:**
**_*E*_, _*Y*_, _*U*_, _*V*_**
**_*Do*_**
Update _*U*_ by (14)
Update _*V*_ by (19)
Update _*E*_ by (10)
Update _μ_ by _μ=ρμ_
Update _*Y*_ by _*Y^r+1^ =Y^r^ +μ^r^ (E^r^ –X+U^r^ (V^r^)^T^)*_
Update _μ_ by _μ^r+1^ =ρμ^r^_
**Until convergence**

## Experiments and Discussion

### Gene Expression Datasets

Five gene expression datasets, which include one benchmark dataset, two single-cancer datasets from TCGA, and two integrated multicancer datasets from TCGA, are used to evaluate the performance of PL21GPCA. The verified experiments consist of two aspects: “tumor sample clustering” and “gene network module discovery.” Based on the optimal low-dimensional (_*k*_-dim) subspace denoted as _*V^T^ =(v_1,⋯,v_n)∈R^k×n^   (V^T^ V=I)*_, the classical clustering method K-means is then used for tumor clustering. For comparison, extensive experiments are also performed using existing dimensionality reduction methods, including SPCA (Journee et al., 2010), RPCA (Candès et al., 2009), gLPCA (Jiang et al., 2013), pgLPCA (Feng et al., 2017) and GNMF (Cai et al., 2011). Among the compared methods, some are based on PCA, and some introduce the graph Laplacian regularization item. Based on the optimal projected matrix *U*_*p×k*_, the differentially expressed genes are selected for gene network analysis to find key genes with close relationships to some cancers.

The details of the five data sets are as follows. The benchmark gene expression dataset is lung cancer data (Bhattacharjee et al., 2001) that have often been employed by researchers to evaluate their algorithms (Lee et al., 2010; Kong et al., 2017), consisting of 12,625 genes of 56 samples. There are four types of lung cancer in the 56 samples: pulmonary carcinoid (20), colon metastases (13), small cell lung carcinoma samples (6) and normal lung samples (17). The two single-cancer datasets and the two integrated multicancer datasets are all from The Cancer Genome Atlas (TCGA) which is known as the largest tumor specimens database. The genomic data provided by TCGA include DNA methylation, microRNA expression, gene expression, protein expression, and DNA copy number, etc. We downloaded gene expression datasets (at level 3) of five different cancers from TCGA: colorectal cancer (CRC), cholangiocarcinoma (CHOL), squamous cell carcinoma of head and neck (HNSC), pancreatic cancer (PAAD), and esophageal cancer (ESCA). Each dataset consists of 20,502 genes expressed in different numbers of samples. In our experiments, CRC and CHOL are used as single-cancer datasets to evaluate the performance of the PL21GPCA method. There are 281 samples for CRC and 45 for CHOL. Each of these two datasets contains two types of cancer samples, “negative” and “positive.” “Negative” or “NT” represents normal samples. “Positive” or “TP” represents diseased samples. There are 262 “TP” samples in the CRC data and 36 in the CHOL data, and the rest are “NT” samples. Two integrated datasets are used to further verify the performance of the PL21GPCA method. Each integrated dataset consists of 3 types of cancers. One of the integrated datasets, H_C_P, contains 836 “TP” samples, among which the sample numbers of the three cancers are 398 (HNSC), 262 (CRC), and 176 (PAAD). The other integrated dataset, E_C_C, contains 481 “TP” samples, in which the sample numbers of the three cancers are 183 (ESCA), 36 (CHOL), and 262 (CRC). The statistics of these datasets are summarized in Table 1.

**TABLE 1:** Statistical information on the experimental data.

**Dataset**		**# of genes (_*p*_)**	**# of samples (_*n*_)**	**# of classes (_*k*_)**
Benchmark Data	Lung Cancer	12625	56	4
Single-Cancer Data from TCGA	CRC	20502	281	2
	CHOL	20502	45	2
Integrated Cancer Data from TCGA	H_C_P	20502	836	3
	E_C_C	20502	481	3

### Tumor Sample Clustering

#### Evaluation Metric

Based on the optimal principal directions *Z*^*T*^ = (*v*_1_,⋯,*v*_*n*_) ∈ *R*^*k*×*n*^ (*Z*^*T*^*V* = *I*), the K-means algorithm is then employed for tumor sample clustering. The accuracy (ACC) and the normalized mutual information (NMI) are the two most commonly used metrics to evaluate the clustering results (Cai et al., 2005). For the _*i*_th sample, we use *p*_*i*_ to denote the prior label and *r*_*i*_ to denote the obtained clustering label. The metric ACC is defined as follows:

(20) $$A\,C\,C=\frac{\sum_{i=1}^{n}\theta \,\left(p_{i},m\,a\,p\,\left(r_{i}\right)\right)}{n},$$

where _*n*_ denotes the total number of samples in every dataset. The function θ(*x*,*y*) equals 1 if *x=y* and 0 otherwise. The function *map*(*r*_*i*_) maps each obtained cluster label *r*_*i*_ to the equivalent prior label. Let _*C*_ be the prior set of clusters and *C*′ be the obtained set from our algorithm. Define their mutual information metric *MI*(*C*,*C*′) as:

(21) $$M\,I\,\left(C,C^{\prime }\right)=\sum p\,\left(c_{i},c_{j}^{\prime }\right)\,\log_{2}\frac{p\,\left(c_{i},c_{j}^{\prime }\right)}{p\,\left(c_{i}\right)\,\cdots \,p\,\left(c_{j}^{\prime }\right)}$$

where *p*(*c*_*i*_) and $p\,\left(c_{j}^{\prime }\right)$are the probabilities that a sample arbitrarily selected from the dataset belongs to clusters *c*_*i*_ and $c_{j}^{\prime }$, respectively, and $p\,\left(c_{i},c_{j}^{\prime }\right)$ is the joint probability. In the experiments, the metric NMI is defined as follows:

(22) $$N\,M\,I\,\left(C,C^{\prime }\right)=\frac{M\,I\,\left(C,C^{\prime }\right)}{\max(H\left(C\right),H\left(C^{\prime }\right)}$$

where *H*(*C*) and *H*(*C*′)are the entropies of _*C*_ and _*C’*_, respectively. Therefore, the metric *NMI*(*C*,*C*′) ranges from 0 to 1. *NMI=1* if the two sets of clusters are identical, and if the two sets are independent, *NMI=0*.

However, a problem that needs to be resolved is that the K-means algorithm may or may not converge to the same solution in each run with random initial conditions. Therefore, the evaluated metrics ACC and NMI obtained by only once-running of k-means is not enough to explain the result. To solve this problem, for the given cluster number _*k*_, K-means was run 50 times on each dataset, and the average performance was computed. As a reference, we also recorded the maximum values of ACC and NMI of the 50 runs. Thus, four metrics, ACC_max, ACC_mean, NMI_max and NIM_mean, are used to evaluate our experiments. Generally, the larger the mean value is, the better is the clustering performance, and the better are the stability and robustness of the clustering. This also indicates that the corresponding dimension reduction method has good robustness and sparse effect.

#### Parameter Selection

**FIGURE 2:**
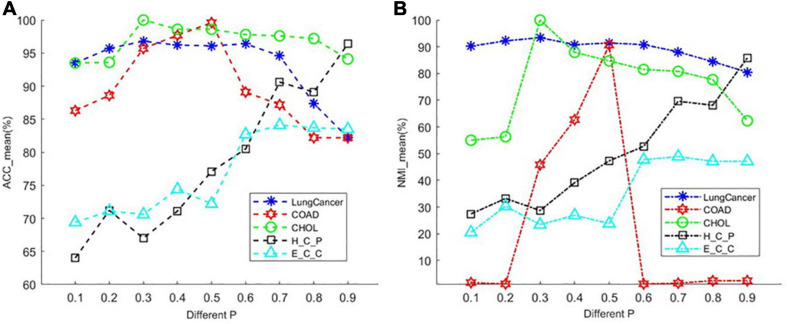
The average performance taking the essential parameter at nine different values from 0.1 to 0.9. **(A)** The mean value of ACC for different cancer datasets. **(B)** The mean value of NMI for different cancer datasets.

The PL21GPCA model has three essential parameters, _*p*_, _λ_, and _α_, which need to be determined in (5). The range of each parameter is 0 < *p* < 1, λ > 0, α > 0. When determining the optimal value of one parameter, the other two parameters are fixed. We focus on the influence of the value of _*p*_ on the performance. PL21GPCA achieves consistently good performance when the two regularization parameters _λ_ and _α_ are varied from 10 to 1,000 on all three datasets. Figure 2 shows how the average performance varies when taking the essential parameter _*p*_ at nine different values from 0.1 to 0.9. For every dataset, extensive experiments are carried out to seek the appropriate parameters to achieve the best performance for tumor sample clustering. Thus, different parameters are chosen for different datasets (see Table 2).

**TABLE 2:** Values of the three parameters _*p*_, _λ_, and _α_ for different datasets.

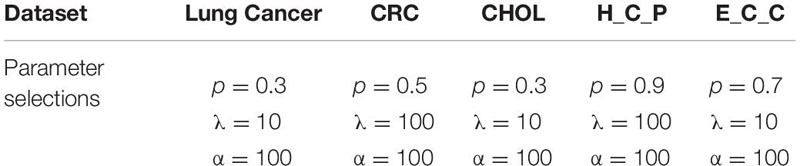

There is another parameter that is not appear in the objective function of PL21GPCA. However, it is also an important parameter affecting the performance of our method. It is parameter *k*′, the number of nearest neighbors of every point when constructing the graph in the step of graph Laplacian embedding. Setting this parameter too small may cause overfitting, and too large may increase the error. By extensive experiments, we find that the appropriate value for this parameter is near the square root of the sample number for different datasets.

#### Clustering Results

Tables 3–5 show the clustering results on the lung cancer data, single-cancer data from TCGA (CRC and CHOL datasets), and integrated cancer data (H_C_P and E_C_C datasets), comparing the PL21GPCA-based method with the competitors. For each dataset with a given cluster number _*k*_, the K-means algorithm was run 50 times to randomize the experiments. The maximum and the mean value metrics are all presented in the tables. The performance of the PL21GPCA-based method is highlighted in bold in the tables. Regardless of the datasets, the PL21GPCA-based method always results in the best performance on the mean value metrics ACC_mean and NMI_mean. As mentioned above, the mean value is more meaningful than the maximum value, which is for reference only. By leveraging the power of three measures, including taking the proximal Lp-norm ∥g∥_*p*_(0 < *p* < 1) on the loss function, employing the L_2,1_-norm regularization item to insure feature selection, and introducing the Laplacian regularization item to emphasize the geometrical structure of the data, the PL21GPCA-based method can always get a better clustering performance.

**TABLE 3:** Clustering performance on lung cancer.

**Methods**	**ACC (%)**		**NMI (%)**	
	**ACC_Max**	**ACC_mean**	**NMI_Max**	**NMI_mean**
SPCA	100	84.39	100	83.07
RPCA	*100*	*86.25*	*100*	*84.77*
GNMF	85.71	79.71	75.57	69.62
gLPCA	89.29	78.5	80.82	69.86
pgLPCA	100	82	100	80.05
PL21GPCA	**100**	**96.8*2***	**100**	**93.44**

**TABLE 4:** Clustering performance on CRC and CHOL.

**Data**	**Method**	**ACC (%)**		**NMI (%)**	
		**ACC_Max**	**ACC_mean**	**NMI_Max**	**NMI_mean**
CRC	SPCA	92.17	87.57	35.3	22.57
	RPCA	*98.22*	*67.95*	*69.82*	*24.33*
	GNMF	88.61	60.5	30.79	18.93
	gLPCA	90.75	87.01	22.7	15
	pgLPCA	94.31	78.65	42.67	20.1
	PL21GPCA	**99.64**	***99.64***	**90.55**	***90.55***
CHOL	SPCA	100	93.38	100	60.65
	RPCA	*100*	*100*	*100*	*100*
	GNMF	100	100	100	100
	gLPCA	100	78.04	100	54.87
	pgLPCA	100	81.87	100	59.83
	PL21GPCA	**100**	**100**	**100**	**100**

**TABLE 5:** Clustering performance on H_C_P and E_C_C.

**Data**	**Method**	**ACC (%)**		**NMI (%)**	
		**ACC_Max**	**ACC_mean**	**NMI_Max**	**NMI_mean**
H_C_P	SPCA	55.26	51.82	17.85	14.98
	RPCA	*91.87*	*77.3*	*71.43*	*68*
	GNMF	57.3	54.02	29.59	22.59
	gLPCA	55.62	52.96	29.43	16.89
	pgLPCA	86.96	70.26	58.42	45.4
	PL21GPCA	**96.41**	**96.41**	**85.77**	**85.75**
E_C_C	SPCA	71.52	67.9	19.28	15.06
	RPCA	*81.08*	*76.17*	*55.72*	*32.47*
	GNMF	68.4	62.05	19.03	9.29
	gLPCA	70.69	69.58	23.14	19.7
	pgLPCA	79.63	72.72	41.33	31.35
	PL21GPCA	**85.65**	**84.09**	**60.31**	**47.15**

For the different types of data used in the experiments, a number of meaningful points need to be emphasized further.

##### The benchmark data

For the lung cancer dataset, Table 3 shows that the PL21GPCA-based method achieves the same performance as SPCA, RPCA and pgLPCA considering the maximum value metrics (the ACC_max and the NIM_max are also 100%) but is obviously superior to the other methods in terms of the mean value metric (ACC_mean reaches 96.82% and the NIM_mean reaches 93.44%).

##### Single-cancer data from TCGA

Table 4 shows the clustering performance of the two single-cancer datasets from TCGA. For the CRC dataset, our method presents very superior performance compared with other methods, with the ACC_mean reaching 99.64% as well as the ACC_max. The good average performance shows the robustness of the PL21GPCA method. In addition, the two NMI metrics (all reaching 90.55%) also go far beyond the performance of other methods. For the CHOL dataset, all the methods achieve the same results (100%) when considering the maximum value metrics. Our method achieves the same performance (100%) as GNMF and RPCA in terms of the mean value metrics. A surmise is reported that there may be distinct discriminations for the two kinds of samples in the original CHOL data (Kong et al., 2017).

##### Integrated multicancer data from TCGA

Table 5 reports the estimation results on the two integrated datasets. It shows that the PL21GPCA method performs much better than the competitors. As highlighted in bold in Table 5, for H_C_P data, the ACC_max and the ACC_mean all reach 96.41%, and the NMI_max and the NMI_mean are also superior to the corresponding values for other methods. For E_C_C data, our method is still outstanding; taking the ACC metric as an example, the ACC_max reaches 85.65%, and the ACC_mean reaches 84.09%. Based on the excellent performance on these two integrated datasets, should we speculate that the PL21GPCA method is more suitable for learning the compact representation of higher-dimensional and more complex data than its competitors, which needs further verification.

Finally, as we can see from Tables 3–5, among the compared methods, the RPCA method performs second to our method and better than the other competitors, such as SPCA, GNMF, gLPCA, and pgLPCA. The performance of RPCA is in italics in the tables. If the intrinsic geometric structure is introduced to RPCA, will the performance be improved further? This question is also worth further verification.

#### Embedding Evaluation

**FIGURE 3:**
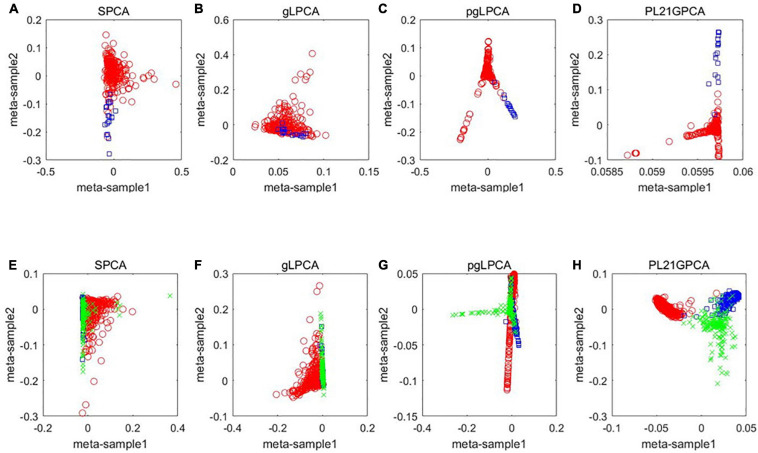
A visualized comparison of low-dimensional embeddings by SPCA, gLPCA, pgLPCA, and PL21GPCA on COAD and H-C-P datasets. **(A–D)** Are the results of the compared methods SPCA, glPCA, gpLPCA, and PL21GPCA respectively on the CRC dataset. **(E–H)** Are the compared results of the four methods on the H-C-P dataset.

To further show the performance of the novel dimensionality reduction method compared others, a visualized data distribution of the low-dimensional embedding corresponding to the first two components of the PCA-based method are demonstrated. Besides the proposed method PL21GPCA, the results of three other methods including SPCA, gLPCA, pgLPCA are compared because these methods are also the direct extensions of PCA. Figure 3 presents the sample clustering results in a two-dimensional space. We choose two representative datasets CRC data and H-C-P data to show the results. Figures 3A–D are the results of the compared methods SPCA, glPCA, gpLPCA and PL21GPCA, respectively, on the CRC dataset. Figures 3E–H are the compared results of the four methods on the H-C-P dataset. No matter for the CRC data which contains two types of cancer samples, or for the H-C-P data which contains three types of cancer samples, SPCA and gLPCA make the samples from different categories being mixed together, and the pgLPCA can only separate the samples into categories roughly, so they have unideal clustering results. However, PL21GPCA make the embeddings of samples in clearer distribution. Therefore, the clustering results is better than the compared methods. The visualized results verified the robustness and the flexibility of the proposed model.

### Experiments on Simulated data

Experiments on simulation data are also carried out to evaluate the effectiveness of PL21GPCA. The simulation data used in the experiment is a matrix *X*_*3000×80*_ generated by *rand* function in Matlab. In order to simulate the representation of features in different types of samples, based on the generated matrix *X*_*3000×80*_, some changes have also been made. Firstly, we add 1 to the values of columns 1 to 20 in rows *i*^∗^30−29(*i* = 1,⋯100) of matrix *X*_*3000×80*_, add 2 to the values of columns 21 to 40 in rows *i*^∗^30−19(*i* = 1,⋯100), add 3 to the values of columns 41 to 60 in rows *i*^∗^30−9(*i* = 1,⋯100), add 4 to the values of columns 61 to 80 in rows *i*^∗^30−5(*i* = 1,⋯100), add 2 to the values of columns 21 to 40 in rows *i*^∗^30−25(*i* = 1,⋯100), add 1 to the values of columns 1 to 20 in rows *i*^∗^30−15(*i* = 1,⋯100), which means that the 80 samples in the simulation data contain four categories. Secondly, we use the function *imnoise* in matlab to add different sizes of Gaussian white noise to *X*. The mean value of the added Gaussian white noise is 0 and the variance _σ^2^_ is chosen in the range of [0.4∼1.2]. Next, we use the proposed method PL21GPCA and the compared methods to reduce the dimension and denoise the simulated data, and then use the K-means method to cluster the denoised data, the evaluation metric ACC_mean mentioned above is used to test the effectiveness of the method. the K-means algorithm is run 50 times to randomize the experiments.

Table 6 shows the experiments results on simulated data. It can be seen evidently that the performances of all methods change with the increase of noise. The best performance of different methods when adding different noises are marked with black bold. Although the performance of pl21GPCA is second only to RPCA when the noise is low (σ^2^ = 0.4), with the increase of Gaussian white noise, the effect of our proposed method is mostly ahead of other methods especially when σ^2^ = 0.6, 0.8, 1.2, which shows that the new method has better de-noising ability and robustness.

**TABLE 6:** Clustering performance on simulated data with different Gaussian white noise.

**Simulated data**	**SPCA**	**RPCA**	**GNMF**	**gLPCA**	**gpLPCA**	**PL21GPCA**
**_σ^2^ =0.4_**	96.6	**99.75**	95.35	87.37	89.47	99.45
**_σ^2^ =0.6_**	94.35	91.33	94.35	84.68	86.45	**97.45**
**_σ^2^ =0.8_**	85.87	91.1	93.85	83.2	85.57	**94.35**
**_σ^2^ =1.0_**	80.12	90.85	**93.4**	86.48	85.67	93.33
**_σ^2^ =1.2_**	70.25	76.43	73.58	85.83	82.12	**87.15**

### Gene Network Module Discovery

Due to the outstanding performance of our method on the CRC dataset and the integrated H_C_P dataset, the construction and analysis of the gene network are based on these two datasets. The strategy of gene network module discovery involves two steps. First, the genes for constructing the co-expression gene networks are selected. Second, based on the filtered genes, co-expression networks are established, and then the key genes that may be closely related to some cancers are analyzed.

#### Gene Selection

In this step, there are two problems to be solved: one is how to select genes, and the other is how many to select. It is known that among thousands of genes, only a handful of them regulate a specific biological process (Delbert et al., 2005; Liu et al., 2013). These minority of genes are called differentially expressed genes (Liu J. et al., 2015). In this article, the differentially expressed genes are selected to carry out gene network analysis according to the projected matrix *U*_*p×k*_. Now, we mark the optimal projected matrix *U*_*p×k*_ as $\tilde{U}$; therefore, these differentially expressed genes can be identified according to $\tilde{U}$ (Liu J. et al., 2015; Feng et al., 2016). We denote $\tilde{U}$as follows:

(23) $$\tilde{U}=\left[\begin{array}{c} {\tilde{u}}_{11}\,\,{\tilde{u}}_{12}\,\,\cdots \,\,{\tilde{u}}_{1\,k}\\ {\tilde{u}}_{21}\,\,{\tilde{u}}_{22}\,\,\cdots \,\,{\tilde{u}}_{2\,k}\\ \,\vdots \,\vdots \,\ddots \,\vdots \,\\ {\tilde{u}}_{p\,1}\,\,{\tilde{u}}_{p\,2}\,\,\cdots \,\,{\tilde{u}}_{p\,k} \end{array}\right]\,$$

The upregulated genes are reflected by the positive value in the matrix $\tilde{U}$, and the downregulated genes are reflected by the positive value (Liu et al., 2013). Therefore, the absolute value of the items in $\tilde{U}$ is used to identify the differentially expressed genes. The items of each row in $\tilde{U}$ are summed, and then the evaluating vector denoted as $\hat{U}$ is obtained:

(24) $$\hat{U}={\left[\sum_{j=1}^{k}{\tilde{u}}_{1\,j}\,\,\sum_{j=1}^{k}{\tilde{u}}_{2\,j}\,\,\cdots \,\,\sum_{j=1}^{k}{\tilde{u}}_{p\,j}\right]}^{T}$$

The larger item in $\hat{U}$ indicates the more strongly differentially expressed gene. Therefore, we sort the elements in $\hat{U}$ in descending order and take the top *l*(*l*≪*p*) elements. In many studies, it has been unclear how many genes should be selected for gene network analysis. Since only a small number of genes can regulate a specific biological process, these genes may play a decisive role in the clustering results of tumor samples. In this paper, the number of genes used for constructing the gene network is determined according to the clustering performance based on the selected genes. Through experimentally investigating the clustering performance with the number of selected genes varied from 500 to 2000, it is found that the clustering results corresponding to **1600** genes are best for the CRC data and **700** for the H_C_P data.

#### Construction of Gene Networks

Suppose *l* differentially expressed genes are used to construct the gene network. Let matrix *R*_*l×n*_ denote the *l* gene expression in _*n*_ samples. We use the Pearson correlation coefficient (PCC) (Hou et al., 2019) to measure the correlation of any two genes in *R*_*l×n*_. The values in the PCC matrix vary in the range of [0,1]. The larger the PCC value is, the higher the correlation is. Based on matrix *R*_*l×n*_, an adjacency matrix *A*_*l×l*_ can be calculated. According to the adjacency matrix, an intuitive visualized graph of the gene interaction network composed of several modules is obtained.

#### Analysis of Gene Network Modules

**FIGURE 4:**
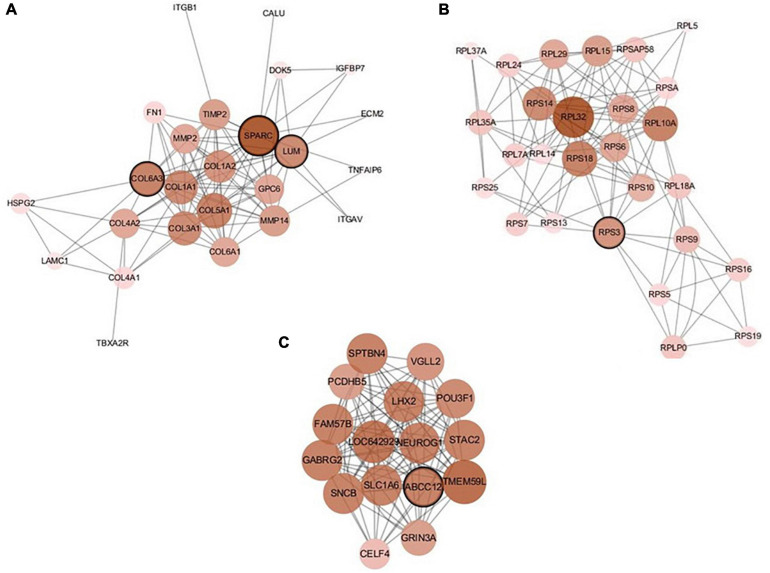
The first three modules of the constructed network based on the CRC data. The five marked genes SPARC, ABCC12, COL6A3, LUM, and RPS3 have been confirmed to be associated with CRC and other cancers. **(A)** Module 1; **(B)** Module 2; **(C)** Module 3.

There are 39 modules, including 218 nodes and 504 edges, in the constructed network based on the CRC data. We analyzed the top 10 nodes (genes) with higher degrees in the first three modules that retained more relevant interactions. The degree of the node (gene) shows its role in the network modules. The larger the degree of the node (gene) is, the more important the node (gene) is, and such nodes (genes) may retain the tight connectivity of the network. Figure 4 shows the main part of the first three gene network modules in which a small number of nodes whose degree is very low have been removed. The roles of the top ten genes in the first three modules are illustrated in Figure 4. The degree value of a node in Figure 4 is represented by its size and color. The larger the node is, the darker its color is, which corresponds to a larger degree of the node. Referring to GeneCard with its website http://www.genecards.org/, we list the annotations of the top ten genes in Table 7. Five of the top ten genes have been validated as associated with multiple cancers: SPARC, ABCC12, COL6A3, LUM, and RPS3. The corresponding nodes of these genes are marked with a black outline in Figure 4 and are also shown in bold in Table 7. In the literature (Liu Q. Z. et al., 2015), the gene SPARC has been recommended as a predictor of colorectal cancer. The gene ABCC12 is a human ATP binding cassette (ABC) transporter and is a multidrug resistance protein (MRP9). However, MRP9 has been recognized as an important target for the immunotherapy of breast cancer (Bera et al., 2002). Studies have shown that colorectal cancer can be predicted by the gene COL6A3 because it is overexpressed in samples of colorectal cancer. Therefore, COL6A3 is considered a potential diagnostic and prognostic marker gene for colorectal cancer (Qiao et al., 2015). As one of the members of the leucine-rich proteoglycan family, the gene Lumican (LUM) is overexpressed in many kinds of cancers, including colorectal, neuroendocrine, cervical, carcinoid, breast, and pancreatic cancer. LUM also causes the growth and invasion of pancreatic cancer (Ishiwata et al., 2007). The ribosomal protein gene S3 (RPS3) is also overexpressed in colorectal cancer. Researchers found an increase in ribosome synthesis in patients with colorectal cancer (Pogue-Geile et al., 1991). Although the other five genes RPL32, TMEM59L, LOC642929, LHX2, and TLCD3B have not been identified in clinical studies indicating their effect on cancers, they may be considered candidate oncogenes because of their high ranking in our constructed gene network modules. By constructing co-expression gene network modules based on the CRC dataset, we found some disease-causing genes for colorectal cancer and other related cancers. It shows that constructing gene network modules via the genes filtered based on PL21GPCA can help us discover the key oncogenes.

**TABLE 7:** Annotations of the top ten genes in the first three network modules based on CRC data.

**Gene**	**Summary**
RPL32	A protein coding gene. Diseases associated with RPL32 include frontal convexity meningioma and retinitis pigmentosa 49
**SPARC**	Diseases associated with SPARC include osteogenesis imperfecta, type xvii and osteogenesis imperfecta, type iv
TMEM59L	TMEM59L (Transmembrane Protein 59 Like) is a protein coding gene. An important paralog of this gene is TMEM59
LOC642929	LOC642929 (General Transcription Factor II, I Pseudogene) is a pseudogene
**ABCC12**	Diseases associated with ABCC12 include familial cold autoinflammatory syndrome 1 and episodic kinesigenic dyskinesia 1. An important paralog of this gene is ABCC11
**COL6A3**	A protein coding gene. An important paralog of this gene is COL6A6
**LUM**	Among its related pathways are defective ST3GAL3, which causes MCT12 and EIEE15, and keratin sulfate/keratin metabolism
LHX2	LHX2 (LIM Homeobox 2) is a protein coding gene. Diseases associated with LHX2 include schizencephaly and retinitis pigmentosa
TLCD3B	TLCD3B (TLC Domain Containing 3B) is a protein coding gene. An important paralog of this gene is TLCD3A
**RPS3**	Diseases associated with RPS3 include eumycotic mycetoma and Waardenburg syndrome, type 3

**FIGURE 5:**
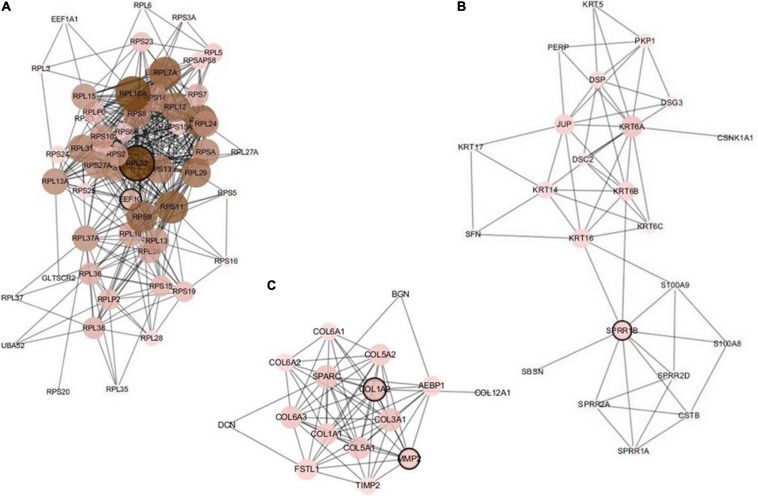
The first three modules of the constructed network based on the H_C_P data. The five marked genes RPL32, EEF1G, SPRR1B, COL1A2, and MMP2 have been confirmed to be associated with multiple cancers. **(A)** Module 1; **(B)** Module 2; **(C)** Module 3.

The constructed network based on the integrated data H_C_P includes 157 nodes and 644 edges. We analyzed the five important nodes (genes) with higher degrees in the first three modules that retained more relevant interactions. Figure 5 illustrates the main part of the first three gene network modules in which the nodes of very low degree have also been removed. Referring to GeneCards, their annotations are listed in Table 8. The five genes RPL32, EEF1G, SPRR1B, COL1A2, and MMP2 have been recognized to be related to multiple cancers. The corresponding nodes of these genes are marked with a black outline in Figure 5. Wan et al. (2004) conducted large-scale experiments on human liver cancer cells. Research has shown that RPL32 is one of the potential genes that affect human cell growth and cancer formation and provides an important tool for diagnostic markers and drug targets (Wan et al., 2004). EEF1G has been thought to be a characteristic gene for colorectal cancer; it is highly expressed in most colorectal cancers and could be considered a marker gene for colorectal cancer detection (Matassa et al., 2013). In addition, the expression level of EEF1G in pancreatic tumor cells was higher than that in normal cells (Lew et al., 1992). SPRR1B is overexpressed in human oral squamous cells. It has been experimentally proven that SPRR1B overexpression in cells will signal MAP kinases but inhibit MAP kinase signals, so SPRR1B can affect cell growth and maintenance (Michifuri et al., 2013). Kiyoshi Misawa and other researchers mainly studied the expression of COL1A2 in head and neck squamous cell carcinoma (HNSC) and found that hypermethylation of CpG may cause inactivation of the gene COL1A2. Therefore, the COL1A2 gene may affect the formation and development of HNSC and could become a major biomarker (Misawa et al., 2011). As a member of the matrix metalloproteinase (MMP) gene family. MMP2 is relevant to the generation of malignant tumors, including colorectal cancer, lung cancer, and breast cancer (Yu et al., 2002; Arajo et al., 2015; Ren et al., 2015). Analysis through the gene network constructed based on integrated multicancer data is helpful for mining the interrelationships between different cancers and genes. It may provide an important reference for the diagnosis and treatment of various diseases.

**TABLE 8:** Annotations of the most important five genes in the first three network modules based on H_C_P data.

**Gene**	**Summary**
RPL32	A protein coding gene. Diseases associated with RPL32 include frontal convexity meningioma and retinitis pigmentosa 49
EEF1G	Diseases associated with EEF1G include gastrointestinal carcinoma. Among its related pathways are viral mRNA translation and gene expression
SPRR1B	A protein coding gene. An important paralog of this gene is SPRR1A
COL1A2	Among its related pathways are ERK signaling and IL4-mediated signaling events
MMP2	Among its related pathways are direct p53 effectors and development endothelin-1/EDNRA signaling

## Conclusion and Suggestions

In this article, we propose a new dimensionality reduction method named PL21GPCA based on PCA for robust tumor sample clustering and gene network module discovery. Based on the traditional PCA, the non-convex proximal Lp-norm ∥g∥_*p*_(0 < *p* < 1)is applied on the loss function to decrease the sensitivity to outliers and noise. The L_2_,_1_-norm is used on the projected matrix to enhance the sparse gene expression in cancer samples. The graph regularization item is introduced to the optimization model to retain the geometric structure of the data. Five gene expression datasets, including one benchmark dataset, two higher-dimensional single-cancer datasets from TCGA, and two integrated multicancer datasets from TCGA, are used to evaluate the performance of our method. The compared experiments demonstrate that the PL21GPCA method outperforms many existing methods in terms of tumor sample clustering. Moreover, this method is employed to discover gene network modules to find the key genes with close relationships to cancers. The results of our study may be a useful reference for clinical diagnosis.

There are some suggestions for future research. First, in the optimization model of PL21GPCA, the constraint used on the loss function is the non-convex proximal Lp-norm ∥g∥_*p*_(0 < *p* < 1), since Lp-norm minimization can result in a sparser solution than the L_1_-norm and perform better in terms of robustness to outliers than the L_2_-norm. However, in addition to the generalized shrinkage operation proposed by Chartrand (2012), there are some other suggestions to address the Lp-norm (0 < *p* < 1) minimization (Guo et al., 2013; Qin et al., 2013) problems. Therefore, we will continue to explore other solutions to the optimization model with the Lp-norm ∥g∥_*p*_(0 < *p* < 1). Second, we will evaluate the performance of PL21GPCA as a compact representation method combined with other methods, including supervised and unsupervised clustering methods such as spectral clustering, support vector machine (SVM) or their improved versions. Third, as mentioned above, the PL21GPCA method gets especially outstanding performance for processing the integrated data, so we will use the PL21GPCA method to process many other integrated data to verify its performance further.

## Data Availability Statement

Publicly available datasets were analyzed in this study. This data can be found here: The lung cancer data http://www.unc.edu/∼haipeng. The TCGA data http://www.tcga.org/.

## Author Contributions

X-ZK conceived and designed the experiments. YS and X-ZK performed the experiments and contributed to the writing of the manuscript. S-SY, JW, and L-YD analyzed the data. J-XL and C-HZ contributed to reagents, materials, and analysis tools. All authors contributed to the article and approved the submitted version.

## Conflict of Interest

The authors declare that the research was conducted in the absence of any commercial or financial relationships that could be construed as a potential conflict of interest.
